# Polymorphisms of *PRLHR* and *HSPA12A* and risk of gastric and colorectal cancer in the Chinese Han population

**DOI:** 10.1186/s12876-015-0336-9

**Published:** 2015-08-25

**Authors:** Qinghua Su, Yuan Wang, Jun Zhao, Cangjian Ma, Tao Wu, Tianbo Jin, Jinkai Xu

**Affiliations:** 1 Department of General Surgery, the Second Affiliated Hospital, Xi’an Jiaotong University School of Medicine, No. 157 West Fifth Road, Xi’an, Shaanxi 710004 China; 2 Inner Mongolia Medical University, Hohhot, Inner Mongolia 010010 China; 3 National Engineering Research Center for Miniaturized Detection Systems, Xi’an, Shaanxi 710069 China; 4 School of Life Sciences, Northwest University, Xi’an, Shaanxi 710069 China

## Abstract

**Background:**

Gastric and colorectal cancers have a major impact on public health, and are the most common malignant tumors in China. The aim of this research was to study whether polymorphisms of *CHCHD3P1-HSP90AB7P*, *GRID1*, *HSPA12A*, *PRLHR*, *SBF2*, *POLD3* and *C11orf93-C11orf92* genes are associated with the risk of gastric and colorectal cancers in the Chinese Han population.

**Methods:**

We genotyped seven single nucleotide polymorphisms (SNPs) from seven genes. We selected 588 patients with gastric cancer and 449 with colorectal cancer, along with 703 healthy controls. All these SNPs were evaluated using the *χ*2 test and genetic model analysis.

**Results:**

The genotype “A/T” of rs12413624 in *PRLHR* gene was associated with a decreased risk of colorectal cancer in allele model analysis [odds ratio (OR) = 0.81; 95 % confidence interval (CI) = 0.68–0.97; *p* = 0.018] and log-additive model analysis (OR = 0.81; 95 % CI = 0.66–0.98; *p* = 0.032). The genotype “A/G” of rs1665650 in *HSPA12A* gene was associated with a decreased risk of gastric cancer in overdominant model analysis (OR = 0.77; 95 % CI = 0.60–0.99; *p* = 0.038).

**Conclusions:**

Our results provide evidence that variants of *PRLHR* gene are a protective factor in colorectal cancer and variants of *HSPA12A* gene are a protective factor in gastric cancer in the Chinese Han population.

## Background

Gastric and colorectal cancers are two of the most widespread cancers worldwide [1]. Both gastrointestinal malignancies are leading causes of cancer-related death in East Asia, Eastern Europe, parts of Central and South America. With improvements in the standard of living and changes in lifestyle, food and the environment, the incidences of gastric and colorectal cancers are constantly increasing in China, where they are now the third most frequent malignancies [2, 3].

In the present study, the low-risk susceptibility markers were previously reported in genome-wide association studies as being related to the risk of digestive system cancer: rs10795668 (10p14), rs10788473 (10q23.1), rs1665650 (10q25.3), rs12413624 (10q26.11), rs10500715 (11p15.4), rs3824999 (11q13.4) and rs3802842 (11q23.1) [4–7].

The prolactin releasing hormone receptor (PRLHR), also known as G-protein-coupled receptor 10, is the receptor for prolactin releasing peptide (PrRP). Numerous studies suggest digestive disease was associated with regulation of feeding and a pivotal role of PrRP in the homeostatic regulation of feeding and energy balance [8]. Evidence from our group has shown that central administration of PrRP decreases feeding and body weight gain in rats and mice, without causing adverse effects [9]. HSPA12A is a member of the heat shock protein (HSP) family and a common molecule within cells that act as a chaperone in conditions of stress, including carcinogenesis [10]. Overexpression of HSPA12A might be associated with poor survival in hepatocellular carcinoma. There is a good correlation between the expression of HSPs and the resistance of cancer cells to chemotherapy [11]. *GRID1* gene encodes glutamate receptor δ1, a subunit of glutamate receptor channels that mediate most of the fast excitatory synaptic transmission in the central nervous system and play key roles in synaptic plasticity [12]. *SBF2* gene appears to influence the sorting and degradation of cell surface receptors, such as epidermal growth factor receptor, with resultant alterations in downstream signaling [4].

The aim of this study was to investigate the relationship between *CHCHD3P1-HSP90AB7P*, *GRID1*, *HSPA12A*, *PRLHR*, *SBF2*, *POLD3*, and *C11orf93-C11orf92* genes and susceptibility to gastric and colorectal cancers in the Chinese Han population.

## Methods

### Ethics statement

The protocol in this study conformed to the principles of the Declaration of Helsinki and was ratified by the Ethical Committee of the Second Affiliated Hospital, Xi’an Jiaotong University School of Medicine, China.

### Study population

We recruited 588 patients with gastric cancer and 449 with colorectal cancer between December 2010 and November 2014 from the Department of General Surgery, the Second Affiliated Hospital, Xi’an Jiaotong University School of Medicine. All of the study participants were from the Chinese Han population living in the area of Xi’an. Confirmed cases were patients who were newly diagnosed and histologically confirmed. According to the recruitment and exclusion standards, we surveyed the patients using a self-designed questionnaire including demographic factors such as age, gender, and education, and potential risk factors including smoking, dietary conditions, alcohol consumption, and family history of cancer [13]. The controls were 703 healthy individuals who were selected from June 2011 to October 2014 from the Medical Examination Center, Department of General Surgery, the Second Affiliated Hospital, Xi’an Jiaotong University School of Medicine. The controls were all Chinese Han living in Xi’an city and surrounding area. We excluded patients with chronic diseases of the kidneys, heart, liver and brain. All participants gave signed informed consent prior to participation in the study.

### Genotyping

We genotyped seven single nucleotide polymorphisms (SNPs) with minor allele frequency (MAF) > 5 % in seven genes in the HapMap Asian population. Genomic DNA was stored at −20 °C and was extracted from whole blood by the phenol–chloroform extraction method. Using an extraction kit (GoldMag, China), we isolated DNA from the samples. DNA concentration was measured by spectrometry (DU530 UV/VIS spectrophotometer; Beckman Instruments, Fullerton, CA, USA). We designed the Multiplexed SNP Mass EXTEND assay using Sequenom MassARRAY Assay Design version 4.0 software [14].

### Statistical analysis

The genotype frequencies of each SNP in the control subjects were checked using the Hardy–Weinberg equilibrium (HWE). Power analysis was carried out using the online calculator at http://sampsize.sourceforge.net/iface/s3.html. Data analysis was performed using SPSS version 16.0 statistical package (SPSS, Chicago, IL, USA) and Microsoft Excel. *P* < 0.05 was considered to represent statistical significance. Differences in the distribution were analyzed using logistic regression. The genotype frequencies of cases and controls were calculated using a *χ*2 test [15, 16]. Odds ratios (ORs) and 95 % confidence intervals (CIs) were tested using unconditional logistic regression analysis with adjustment for age and gender [17]. The allele, overdominant and log-additive models were applied using PLINK software (http://pngu.mgh.harvard.edu/purcell/plink/) to assess the association of SNPs with the risk of gastric and colorectal cancers.

## Results

The 588 gastric cancer cases comprised 392 men and 196 women with a mean age of 58.12 ± 11.66 years. The 449 colorectal cancer cases comprised 260 men and 189 women with a mean age of 59.09 ± 11.78 years. The 703 healthy controls comprised 396 men and 307 women with a mean age of 48.57 ± 9.43 years. We found no differences between gender and age distribution. The characteristics of the patients and controls are shown in Table 1. The primers of the seven selected SNPs are shown in Table 2, which were designed by Sequenom MassARRAY Assay Design 4.0 Software [14]. Seven SNPs in seven genes were analyzed in this study. SNP ID, gene, HWE test results, minor/major alleles, and MAF of cases and controls of all the SNPs are shown in Table 3. The minor allele of each SNP, a risk factor, was compared with the wild-type allele.

**Table 1:** Demographic characteristics of patients with gastric and colorectal cancers, and controls

Group (N)	Age (years)	Gender (male/female)	*P* value^a^	*P* value^b^
Healthy controls (*N* = 703)	48.57 ± 9.43	396/307	–	–
Gastric cancer cases (*N* = 588)	58.12 ± 11.66	392/196	0.21	0.54
Colorectal cancer cases (*N* = 449)	59.09 ± 11.78	260/189	0.32	0.25

^a^*P* value is based on the age versus healthy controls in the study

^b^*P* value is based on the gender versus healthy controls in the study

**Table 2:** Primers used for this study

SNP ID	1st – PCR primer sequences	2nd – PCR primer sequences	UEP sequences
rs10795668	ACGTTGGATGAATACTTGTACCTTGGTGGG	ACGTTGGATGTCATCTATGAGCAGCAGCAG	gcGAAAGAGAAAAAGTTAGATTCTTA
rs10788473	ACGTTGGATGCAGGAAGTGACAGCTATCTC	ACGTTGGATGGGCTTCATTGGGAGCTAGTG	ggggaTCCAAGCTACGGCTCACCTGG
rs1665650	ACGTTGGATGCCAACTGAGGATGATTTGAC	ACGTTGGATGGGTTGTTTGGCTACTCAAAG	ctccAAATGTCTATCGCCTTTAC
rs12413624	ACGTTGGATGGCTAGGTGTGGCACTGTTTG	ACGTTGGATGTTATGCAACTGGTCCTGGTC	tgggtTGGTCCTGGTCAGATGTTAT
rs10500715	ACGTTGGATGAGGCTTGAGATTTGGAAGGC	ACGTTGGATGCCATCTTTAGATCTTCTCTC	cttTTTAGATCTTCTCTCAGTCTA
rs3824999	ACGTTGGATGCTAAATCCCCTTTGCTGGAC	ACGTTGGATGGATCAGAGAACTACAAGCAC	TTCTCCATTGGTTCTCTAA
rs3802842	ACGTTGGATGCATCGTTTTGTTAGGAAGAC	ACGTTGGATGGGCCCCTAAAATGAGGTGAA	aagGAGGTGAATTTCTGGGA

*PCR* polymerase chain reaction, *UEP* unextended mini-sequencing primer

**Table 3:** Basic information of candidate SNPs in this study

SNP ID	Gene	HWE *p* value	Alleles A/B	MAF control	MAF case	
					Gastric cancer	Colorectal cancer
rs10795668	*CHCHD3P1–HSP90AB7P*	0.1739	A/G	0.384	0.369	0.348
rs10788473	*GRID1*	0.7497	T/C	0.383	0.391	0.378
rs1665650	*HSPA12A*	1	A/G	0.312	0.316	0.33
rs12413624	*PRLHR*	0.3968	A/T	0.431	0.405	0.381
rs10500715	*SBF2*	0.9056	G/T	0.198	0.212	0.205
rs3824999	*POLD3*	0.7421	C/A	0.361	0.346	0.391
rs3802842	*C11orf92–C11orf93*	0.3986	C/A	0.435	0.441	0.478

A/B stands for minor/major alleles on the control sample frequencies

SNPs are excluded at 5 % HWE *P* level

Further model association analyses used logistic tests including allele model, overdominant model and log-additive model (Table 4). The genotype “A/T” of rs12413624 is associated with a decreased risk of colorectal cancer by allele model analysis (OR = 0.81; 95 % CI = 0.68–0.97; *p* = 0.018) and log-additive model analysis (OR = 0.81; 95 % CI = 0.66–0.98; *p* = 0.032). The genotype “A/G” of rs1665650 was associated with a decreased risk of gastric cancer risk by overdominant model analysis (OR = 0.77; 95 % CI = 0.60–0.99; *p* = 0.038).

**Table 4:** Association of SNPs with risk of gastric and colorectal cancers based on logistic tests adjusted by gender and age

SNP ID	Model	Genotype	Gastric cancer			Colorectal cancer		
			OR (95 % CI)		*P* value	OR (95 % CI)		*P* value
rs10795668	Allele model	A/G	0.94	(0.80–1.10)	0.419	0.86	(0.70–1.02)	0.082
	Overdominant model	A/G	0.97	(0.76–1.23)	0.78	0.89	(0.68–1.17)	0.41
	Log - additive model	–	0.95	(0.80–1.13)	0.56	0.86	(0.71–1.04)	0.12
rs10788473	Allele model	T/C	1.04	(0.88–1.22)	0.655	0.98	(0.82–1.17)	0.817
	Overdominant model	T/C	0.86	(0.68–1.10)	0.24	1.06	(0.81–1.39)	0.66
	Log - additive model	–	1.04	(0.88–1.24)	0.63	0.91	(0.75–1.11)	0.34
rs1665650	Allele model	A/G	1.02	(0.86–1.21)	0.85	1.09	(0.91–1.30)	0.363
	Overdominant model	A/G	0.77	(0.60–0.99)	0.038*	1.04	(0.80–1.36)	0.78
	Log - additive model	–	1	(0.83–1.20)	0.99	1.16	(0.95–1.42)	0.15
rs12413624	Allele model	A/T	0.9	(0.77–1.05)	0.191	0.81	(0.68–0.96)	0.018*
	Overdominant model	A/T	0.91	(0.72–1.17)	0.47	0.9	(0.69–1.18)	0.44
	Log - additive model	–	0.93	(0.77–1.11)	0.39	0.81	(0.66–0.98)	0.032*
rs10500715	Allele model	G/T	1.09	(0.90–1.32)	0.387	1.04	(0.85–1.28)	0.699
	Overdominant model	G/T	1.1	(0.85–1.42)	0.48	0.94	(0.71–1.26)	0.69
	Log - additive model	–	1.07	(0.87–1.32)	0.53	1.05	(0.83–1.32)	0.7
rs3824999	Allele model	C/A	0.94	(0.80–1.10)	0.432	1.14	(0.95–1.35)	0.151
	Overdominant model	C/A	0.81	(0.64–1.04)	0.1	0.98	(0.75–1.28)	0.9
	Log - additive model	–	0.92	(0.77–1.10)	0.37	1.13	(0.93–1.37)	0.21
rs3802842	Allele model	C/A	1.02	(0.88–1.20)	0.774	1.19	(1.00–1.40)	0.541
	Overdominant model	C/A	0.99	(0.77–1.26)	0.91	1.09	(0.84–1.42)	0.52
	Log - additive model	–	0.96	(0.81–1.14)	0.68	1.14	(0.94–1.37)	0.18

*p < 0.05, statistical significance

## Discussion

Gastric and colorectal cancers are the most frequent malignancies diagnosed worldwide and the most common cause of cancer mortality in China [18]. Environmental components are risk factors for the development of gastric and colorectal cancers, such as Helicobacter Pylori infection, salted food intake, changed lifestyle and smoking, and their mortality rates are continually increasing in China [19, 20].

In this study, we showed that the *PRLHR* gene, which is mapped to chromosome 10q26.11, contained an SNP (genotype “A/T” of rs12413624) associated with a increased risk of colorectal cancer. PRLHR is the receptor for PrRP (also known as G-protein-coupled receptor 10) and has pivotal functions in press hormone release and feeding behavior [21]. PrRP, a hormone, may be secreted from peripheral tissues (pancreas, placenta, adrenal) upon the anterior pituitary, or may be secreted from hypophysiotropic neurons by an indirect pivotal mechanism [22]. It was also reported that PRLHR, as well as involvement in the physiological responses to central dministration of PrRP, may play roles in other processes, such as feeding behavior, pathogenesis of uterine fibroids, energy expenditure, obesity and the pivotal control of blood pressure [8, 23, 24]. According to previous reports, rs12413624 is associated with pancreatic ductal adenocarcinoma risk in individuals of European descent but not in Japanese and Chinese populations [25]. We discovered the relationship between rs12413624 in the *PRLHR* gene and colorectal cancer in the allele and the log-additive models. However, we did not find any correlation between the *PRLHR* gene and gastric cancer. It is necessary to study the biological functions of the *PRLHR* gene in further research.

We genotyped “A/G” of rs1665650 in *HSPA12A* gene, which is mapped to chromosome 10q25.3, and associated with a decreased risk of gastric cancer. HSPA12A, heat shock 70-kDa protein 12A, is a novel and atypical member of the HSP70 family in animals. Its effects are diverse and include involvement in the development of atherosclerotic lesions in mice [26]. Cancer cells experience high levels of proteotoxic stress and rely upon stress response pathways for survival and proliferation, thereby becoming dependent on proteins such as stress-inducible HSPs. It is reported that overexpression of HSPA12A in hepatocellular carcinoma tissues is significantly related to poor survival [11]. During carcinogenesis, expression of HSPs is altered in many tumor types. Increased levels of HSPA are related to malignancy, metastasis, poor prognosis, and resistance to therapeutic strategies, including chemotherapy or radiation in glioblastoma, and breast, bladder, endometrial and cervical carcinomas [27–29]. It is also reported that the associations between rs1665650, rs3824999 and colorectal cancer are not strongly modified by gender, alcohol, smoking, aspirin, and various dietary factors [30]. Our results showed that rs1665650 in *HSPA12A* gene is correlated with gastric cancer risk, and we did not find a significant association with the risk of colorectal cancer. Further research should use a larger number of samples and focus on understanding the mechanisms by which *HSPA12A* gene influences pathogenesis and progression.

The rs10795668 in *CHCHD3P1-HSP90AB7P* gene is associated with the risk of colorectal cancer in Poland, Estonia, Lithuania and Latvia [31]. Somatic exonuclease domain mutations in *POLE* gene have been identified in colorectal and endometrial cancer patients, and show an association with hypermutability and microsatellite stability [32]. In both population that included cases of European descent and in a combined analysis with cases from China, SNPs in the *SBF2* gene were associated with survival time among patients with pancreatic adenocarcinoma [7]. However, we did not find that SNPs and genes were associated with gastric or colorectal cancers in our study.

There were several limitations to our study. First, all the samples were from the Chinese Han population living in Xi’an city or its surrounding area and from the same hospital. There were a substantial number of confounding factors that may have caused type I errors (false-positive results) in our association study. Second, we also performed Bonferroni correction of the 21 tests and found no significant results. However, the main weakness of Bonferroni correction is that the interpretation of a finding depends on the number of other tests performed. True important differences may be deemed nonsignificant since the likelihood of type II errors also increased [33]. Finally, our samples included 1037 cases (588 gastric cancer and 449 colorectal cancer) and 703 healthy controls and we performed a power analysis that showed that the power of seven SNPs was < 0.75. The sample size was not large enough for association studies and a larger sample size is required to confirm our findings.

## Conclusions

This study shows that *PRLHR* gene is a protective factor in colorectal cancer and *HSPA12A* gene is a protective factor in gastric cancer. We demonstrated a relationship between polymorphisms of *PRLHR* and *HSPA12A* gene and the risk of gastric and colorectal cancers in the Chinese Han population.

## Abbreviations

PRLHR: Receptor for prolactin releasing peptide
HSPA12A: Heat shock protein 12A
SNPs: Single nucleotide polymorphisms
MAF: Minor allele frequency
HWE: Hardy - Weinberg equilibrium
ORs: Odds ratios
CIs: Confidence intervals
PCR: Polymerase chain reaction
UEP: Unextended mini-sequencing primer

## References

[1] Siegel RL, Miller KD, Jemal A (2015). Cancer statistics, 2015. CA Cancer J Clin.

[2] Akanuma N, Hoshino I, Akutsu Y, Murakami K, Isozaki Y, Maruyama T (2014). MicroRNA-133a regulates the mRNAs of two invadopodia-related proteins, FSCN1 and MMP14, in esophageal cancer. Br J Cancer.

[3] Siegel R, Desantis C, Jemal A (2014). Colorectal cancer statistics, 2014. CA Cancer J Clin.

[4] Wu C, Kraft P, Stolzenberg-Solomon R, Steplowski E, Brotzman M, Xu M (2014). Genome-wide association study of survival in patients with pancreatic adenocarcinoma. Gut.

[5] Fernandez-Rozadilla C, Cazier JB, Tomlinson IP, Carvajal-Carmona LG, Palles C, Lamas MJ (2013). A colorectal cancer genome-wide association study in a Spanish cohort identifies two variants associated with colorectal cancer risk at 1p33 and 8p12. BMC Genomics.

[6] Dunlop MG, Dobbins SE, Farrington SM, Jones AM, Palles C, Whiffin N (2012). Common variation near CDKN1A, POLD3 and SHROOM2 influences colorectal cancer risk. Nat Genet.

[7] Panagiotou OA, Ioannidis JP, Genome-Wide Significance P (2012). What should the genome-wide significance threshold be? Empirical replication of borderline genetic associations. Int J Epidemiol.

[8] Varghese BV, Koohestani F, McWilliams M, Colvin A, Gunewardena S, Kinsey WH (2013). Loss of the repressor REST in uterine fibroids promotes aberrant G protein-coupled receptor 10 expression and activates mammalian target of rapamycin pathway. Proc Natl Acad Sci U S A.

[9] Dodd GT, Luckman SM (2013). Physiological roles of GPR10 and PrRP signaling. Front Endocrinol.

[10] Zhu Y, Ren C, Wan X, Zhu Y, Zhu J, Zhou H (2013). Gene expression of Hsp70, Hsp90 and Hsp110 families in normal palate and cleft palate during mouse embryogenesis. Toxicol Ind Health.

[11] Yang Z, Zhuang L, Szatmary P, Wen L, Sun H, Lu Y (2015). Upregulation of heat shock proteins (HSPA12A, HSP90B1, HSPA4, HSPA5 and HSPA6) in tumour tissues is associated with poor outcomes from HBV-related early-stage hepatocellular carcinoma. Int J Med Sci.

[12] Yamazaki M, Araki K, Shibata A, Mishina M (1992). Molecular cloning of a cDNA encoding a novel member of the mouse glutamate receptor channel family. Biochem Biophys Res Commun.

[13] Wen YY, Pan XF, Loh M, Tian Z, Yang SJ, Lv SH (2012). ADPRT Val762Ala and XRCC1 Arg194Trp polymorphisms and risk of gastric cancer in Sichuan of China. Asian Pac J Cancer Prev.

[14] Trembizki E, Smith H, Lahra MM, Chen M, Donovan B, Fairley CK (2014). High-throughput informative single nucleotide polymorphism-based typing of Neisseria gonorrhoeae using the Sequenom MassARRAY iPLEX platform. J Antimicrob Chemother.

[15] Kochl S, Niederstatter H, Parson W (2005). DNA extraction and quantitation of forensic samples using the phenol-chloroform method and real-time PCR. Methods Mol Biol.

[16] Adamec C (1964). [Example of the use of the nonparametric test. Test X2 for comparison of 2 independent examples]. Ceskoslovenske zdravotnictvi.

[17] Bland JM, Altman DG (2000). Statistics notes. The odds ratio. BMJ.

[18] Atoum MF, Tchoporyan MN (2014). Association between circulating vitamin D, the Taq1 vitamin D receptor gene polymorphism and colorectal cancer risk among Jordanians. Asian Pac J Cancer Prev.

[19] Wan DS (2009). [Epidemiologic trend of and strategies for colorectal cancer]. Ai zheng = Aizheng = Chinese journal of cancer.

[20] D’Elia L, Galletti F, Strazzullo P (2014). Dietary salt intake and risk of gastric cancer. Cancer Treat Res.

[21] Samson WK, Taylor MM (2006). Prolactin releasing peptide (PrRP): an endogenous regulator of cell growth. Peptides.

[22] Morales T, Sawchenko PE (2003). Brainstem prolactin-releasing peptide neurons are sensitive to stress and lactation. Neuroscience.

[23] Samson WK, Resch ZT, Murphy TC (2000). A novel action of the newly described prolactin-releasing peptides: cardiovascular regulation. Brain Res.

[24] Bjursell M, Lenneras M, Goransson M, Elmgren A, Bohlooly YM (2007). GPR10 deficiency in mice results in altered energy expenditure and obesity. Biochem Biophys Res Commun.

[25] Campa D, Rizzato C, Bauer AS, Werner J, Capurso G, Costello E (2013). Lack of replication of seven pancreatic cancer susceptibility loci identified in two Asian populations. Cancer Epidemiol Biomarkers Prev.

[26] Han Z, Truong QA, Park S, Breslow JL (2003). Two Hsp70 family members expressed in atherosclerotic lesions. Proc Natl Acad Sci U S A.

[27] Syrigos KN, Harrington KJ, Karayiannakis AJ, Sekara E, Chatziyianni E, Syrigou EI (2003). Clinical significance of heat shock protein-70 expression in bladder cancer. Urology.

[28] Piura B, Rabinovich A, Yavelsky V, Wolfson M (2002). [Heat shock proteins and malignancies of the female genital tract]. Harefuah.

[29] Thanner F, Sutterlin MW, Kapp M, Rieger L, Kristen P, Dietl J (2003). Heat-shock protein 70 as a prognostic marker in node-negative breast cancer. Anticancer Res.

[30] Kantor ED, Hutter CM, Minnier J, Berndt SI, Brenner H, Caan BJ (2014). Gene-environment interaction involving recently identified colorectal cancer susceptibility Loci. Cancer Epidemiol Biomarkers Prev.

[31] Serrano-Fernandez P, Dymerska D, Kurzawski G, Derkacz R, Sobieszczanska T, Banaszkiewicz Z (2015). Cumulative small effect genetic markers and the risk of colorectal cancer in Poland, Estonia, Lithuania, and Latvia. Gastroenterol Res Pract.

[32] Briggs S, Tomlinson I (2013). Germline and somatic polymerase epsilon and delta mutations define a new class of hypermutated colorectal and endometrial cancers. J Pathol.

[33] Perneger TV (1998). What’s wrong with Bonferroni adjustments. BMJ.

